# Correction: Kim et al. Transcriptomic Analysis of Air–Liquid Interface Culture in Human Lung Organoids Reveals Regulators of Epithelial Differentiation. *Cells* 2024, *13*, 1991

**DOI:** 10.3390/cells15030255

**Published:** 2026-01-29

**Authors:** Jieun Kim, Eun-Young Eo, Bokyong Kim, Heetak Lee, Jihoon Kim, Bon-Kyoung Koo, Hyung-Jun Kim, Sukki Cho, Jinho Kim, Young-Jae Cho

**Affiliations:** 1 Division of Pulmonary and Critical Care Medicine, Department of Internal Medicine, Seoul National University College of Medicine, Seoul National University Bundang Hospital, Seongnam 13620, Republic of Korea; jekim2022@chauniv.ac.kr (J.K.); r0713@snubh.org (E.-Y.E.); r2980@snubh.org (B.K.); dr.hjkim@snubh.org (H.-J.K.); 2 Department of Biomedical Science, CHA University, Seongnam 13488, Republic of Korea; 3 Center for Genome Engineering, Institute for Basic Science, Daejeon 34126, Republic of Korea; leeheetak@ibs.re.kr (H.L.); jhkim@catholic.ac.kr (J.K.); koobk@ibs.re.kr (B.-K.K.); 4 Department of Medical and Biological Sciences, The Catholic University of Korea, Bucheon 14662, Republic of Korea; 5 Department of Thoracic and Cardiovascular Surgery, Seoul National University Bundang Hospital, Seongnam 13620, Republic of Korea; skcho@snubh.org; 6 Department of Genomic Medicine, Seoul National University Bundang Hospital, Seongnam 13620, Republic of Korea; 7 Precision Medicine Center, Future Innovation Research Division, Seoul National University Bundang Hospital, Seongnam 13620, Republic of Korea; 8 Department of Laboratory Medicine, Seoul National University College of Medicine, Seoul National University Bundang Hospital, Seongnam 13620, Republic of Korea

In the original publication [1], corrections were made to Figure 5 and to the title of Section 2.5 in Materials and Methods, and the Informed Consent Statement was added.

## Error in Figure 5

There was a mistake in Figure 5C as published. In Figure 5C, the edge connecting HIF1A and CDKN1A was incorrectly shown as a coherent edge. This edge is not coherent and should be represented as a thin line indicating incoherence. The corrected Figure 5 appears below.

**Figure 5:**
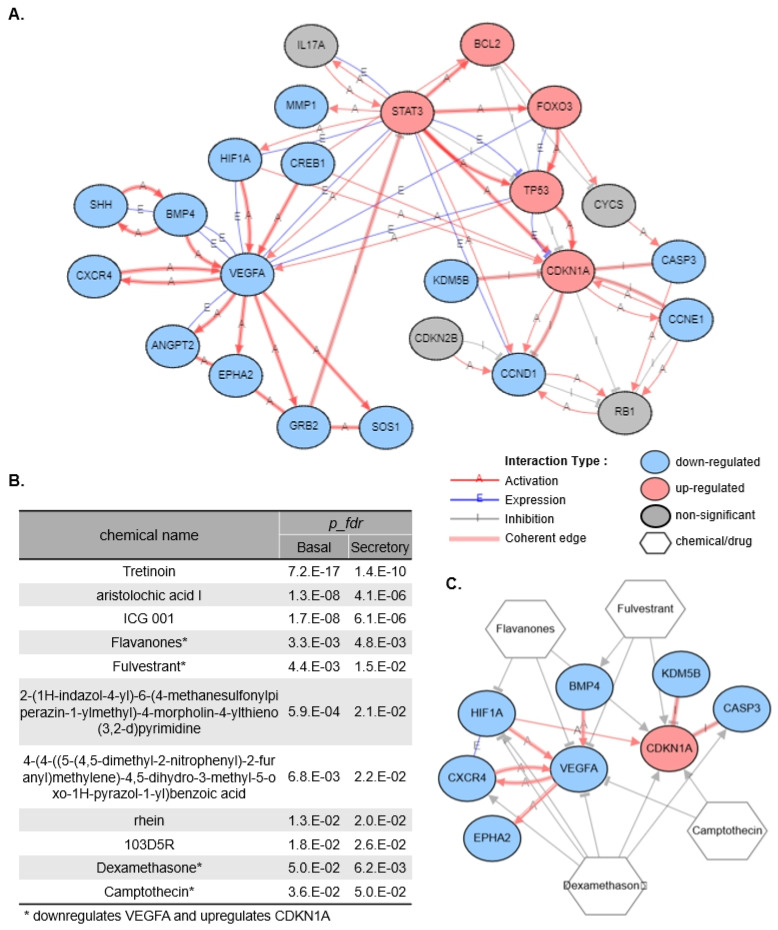
In-silico chemical screening of *VEGFA* inhibitors with *CDKN1A* activation properties. (**A**) The GRN network in Figure 4E was extended by adding genes from the KEGG pathway. (**B**) Upstream chemicals predicted as present in ALIEX basal vs. O1 basal and ALIEX secretory vs. O1 secretory DE results that regulate any of the 8 target genes (*VEGFA*, *CDKN1A*, *HIF1A*, *BMP4*, *CXCR4*, *EPHA2*, *CASP3*, and *KDM5B*). * indicates a chemical that downregulates *VEGFA* and upregulates *CDKN1A*. (**C**) GRN graph summarizing the regulatory effects of selected chemicals on the target genes.

## Text Correction

There was an error in 2. Materials and Methods: the title of Section 2.5 misspelled Psudotime. The corrected title is as follows:

*2.5. RNA Velocity and Ps* *e* *udotime Analyses*

## Missing Informed Consent Statement

The Informed Consent Statement was not stated. The Informed Consent Statement has now been inserted by adding the following Informed Consent Statement section as below.

**Informed Consent Statement:** Informed consent was obtained from all subjects involved in the study.

The authors state that the scientific conclusions are unaffected. This correction was approved by the Academic Editor. The original publication has also been updated.

## References

[1] Kim J., Eo E.-Y., Kim B., Lee H., Kim J., Koo B.-K., Kim H.-J., Cho S., Kim J., Cho Y.-J. (2024). Transcriptomic Analysis of Air–Liquid Interface Culture in Human Lung Organoids Reveals Regulators of Epithelial Differentiation. Cells.

