# Supplementary material for: Transcriptomic Analysis of Air–Liquid Interface Culture in Human Lung Organoids Reveals Regulators of Epithelial Differentiation
Source: Cells. 2024 Dec 2;13(23):1991. doi: 10.3390/cells13231991 (PMC11639892; doi:10.3390/cells13231991)
Supplement: Supplementary file 1 [file cells-13-01991-s001.zip › cells-3271672-supplementary.pdf]

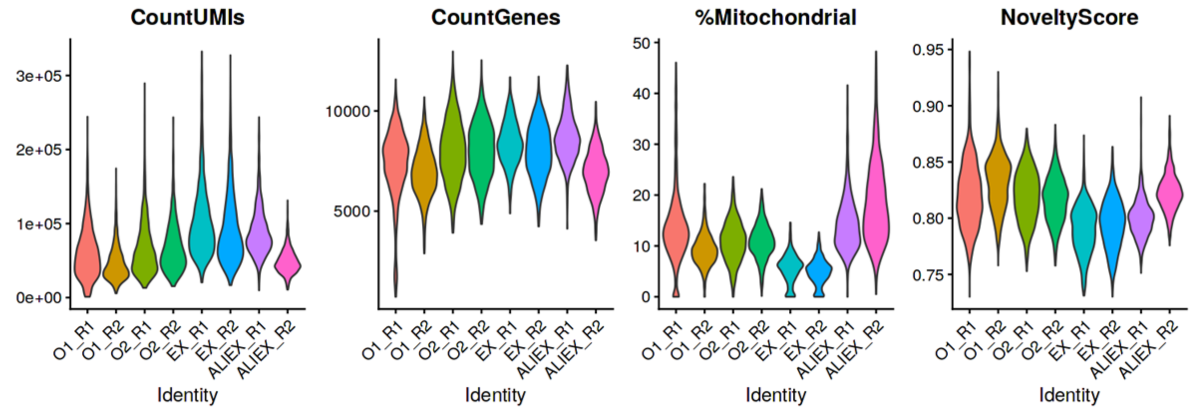

Supplemental Figure S1. Violin plots of unique molecular identifier (UMI) counts (CountUMIs), gene quantity (CountGenes), mitochondrial count ratio (%Mitochondrial), and novelty score (NoveltyScore) for each sample after quality control.

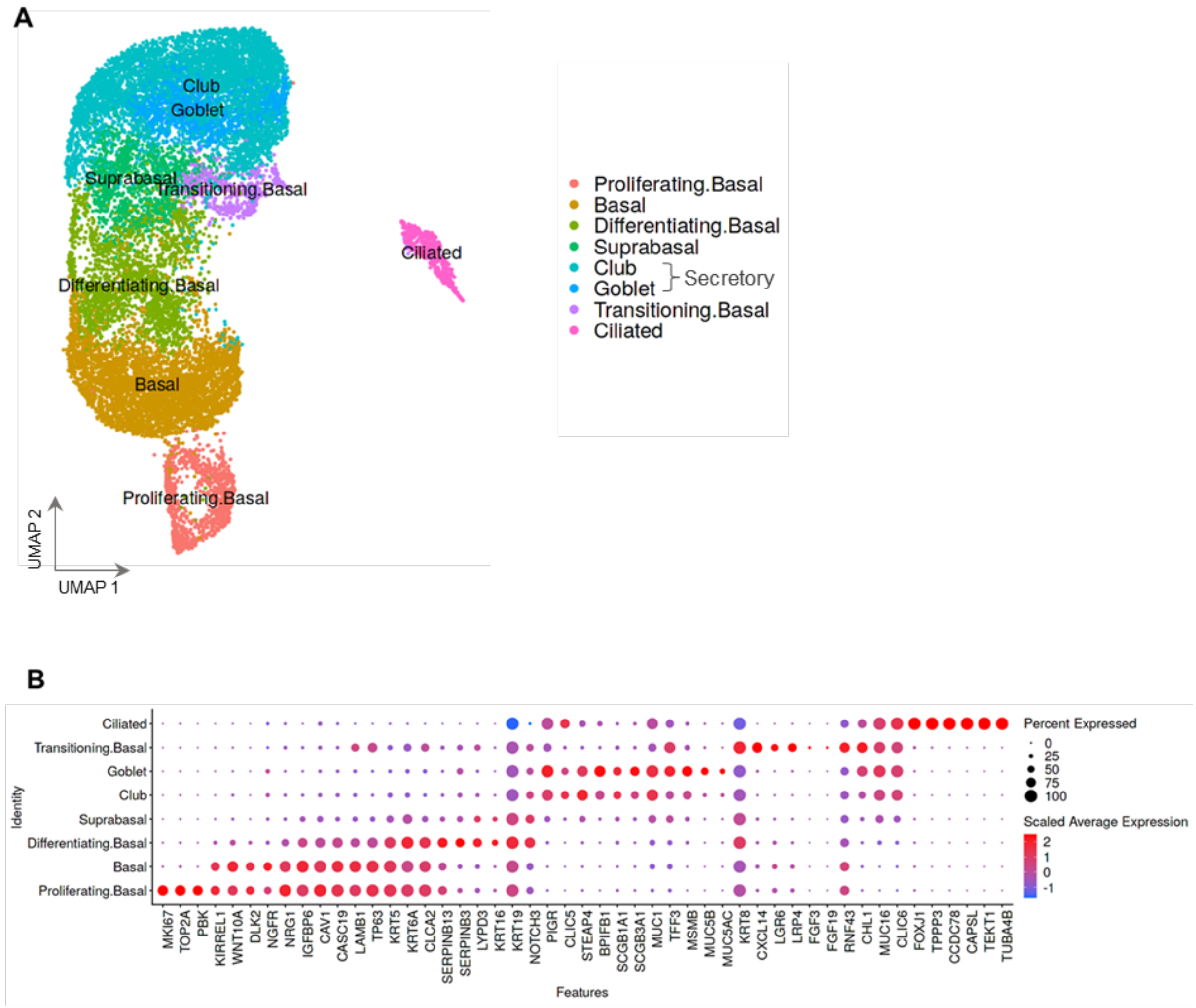

Supplemental Figure S2. Cell Subtypes were identified using known and conserved marker genes. (**A**) Uniform manifold approximation and projection (UMAP) visualization of the entire dataset. Each distinct cell type was defined by a specific color. (**B**) Dot plot showing the scaled average expression values of canonical airway epithelial marker genes for each cluster. The size of the dots is proportional to percent expression with upregulation (red) and downregulation (blue).

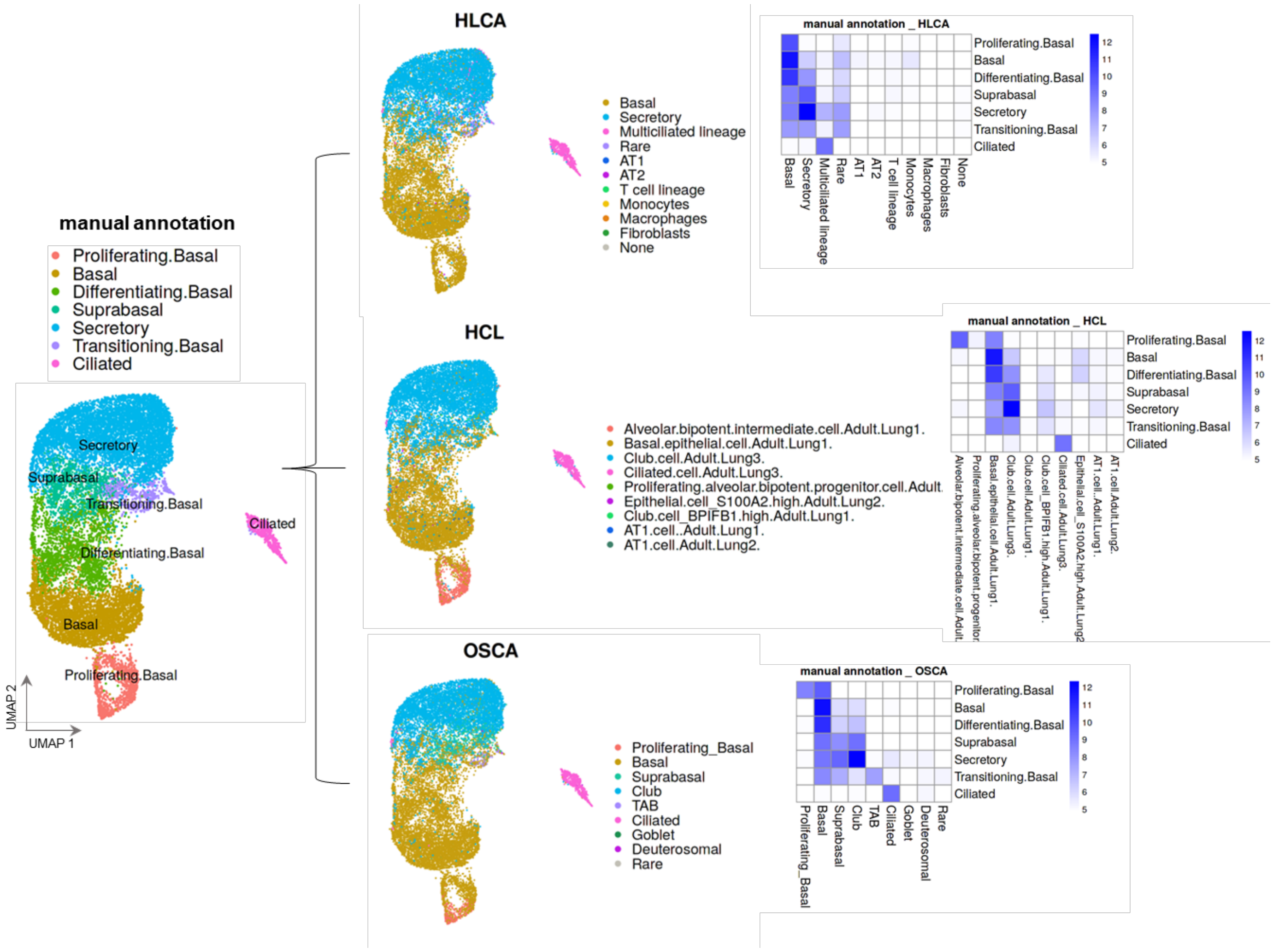

Supplemental Figure S3. Correlation plots of annotated and reference annotated cell types. Three public datasets (HLCA, HCL, and OSCA) were used for validation.

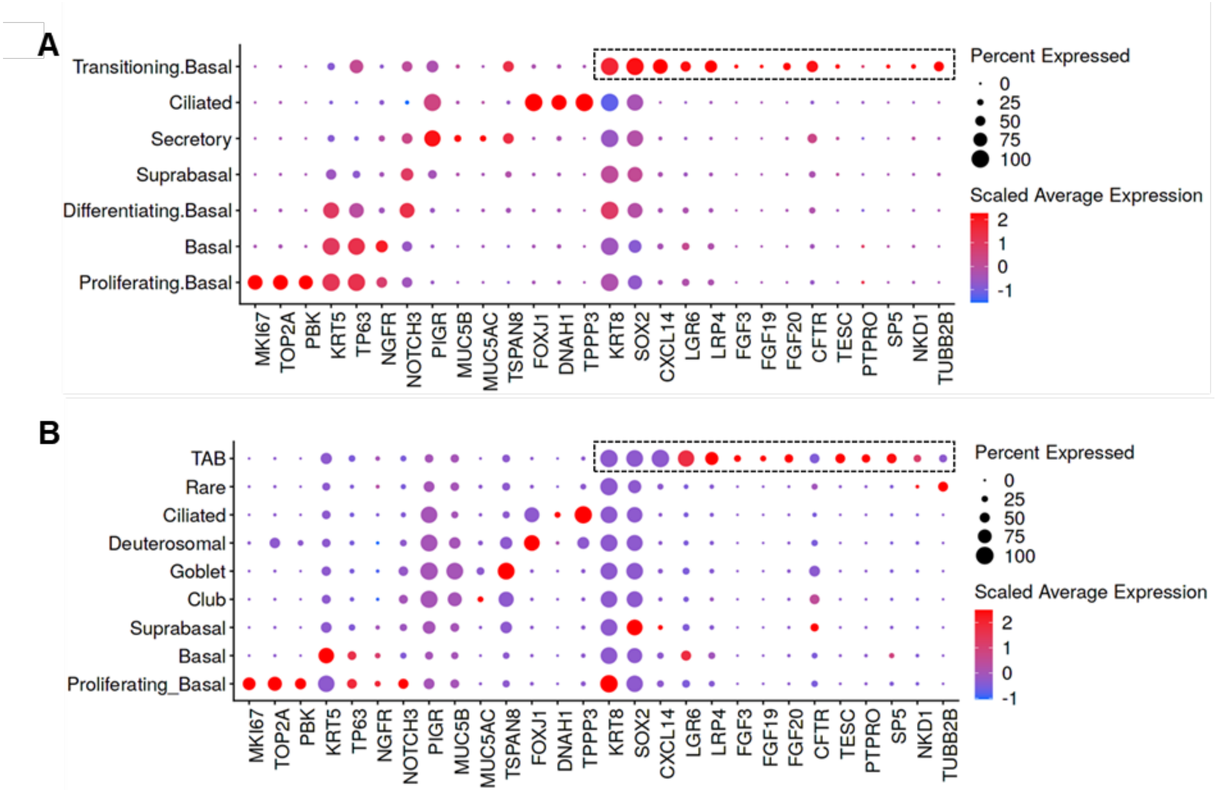

Supplemental Figure S4. Identification of the atypical basal subtype: Transitioning basal. Differences in gene expression profiles between transitioning basal cells in the current dataset (**A**) and TAB (transitioning airway basal) cells in the OSCA dataset (**B**). The size of the dots is proportional to percent expression with upregulation (red) and downregulation (blue).

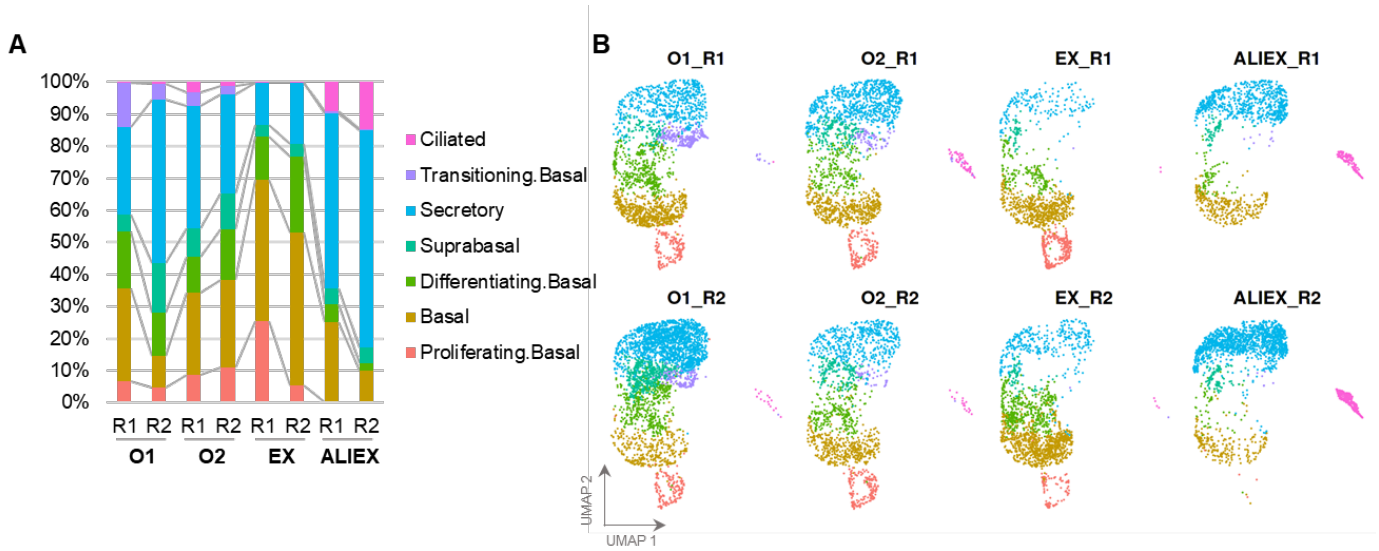

Supplemental Figure S5. Cell composition of each sample. (**A**) Bar graph of subtype compositions (Figure 2B) identified in each sample. (**B**) UMAP visualization of each sample. Each distinct cell type was defined by a specific color.

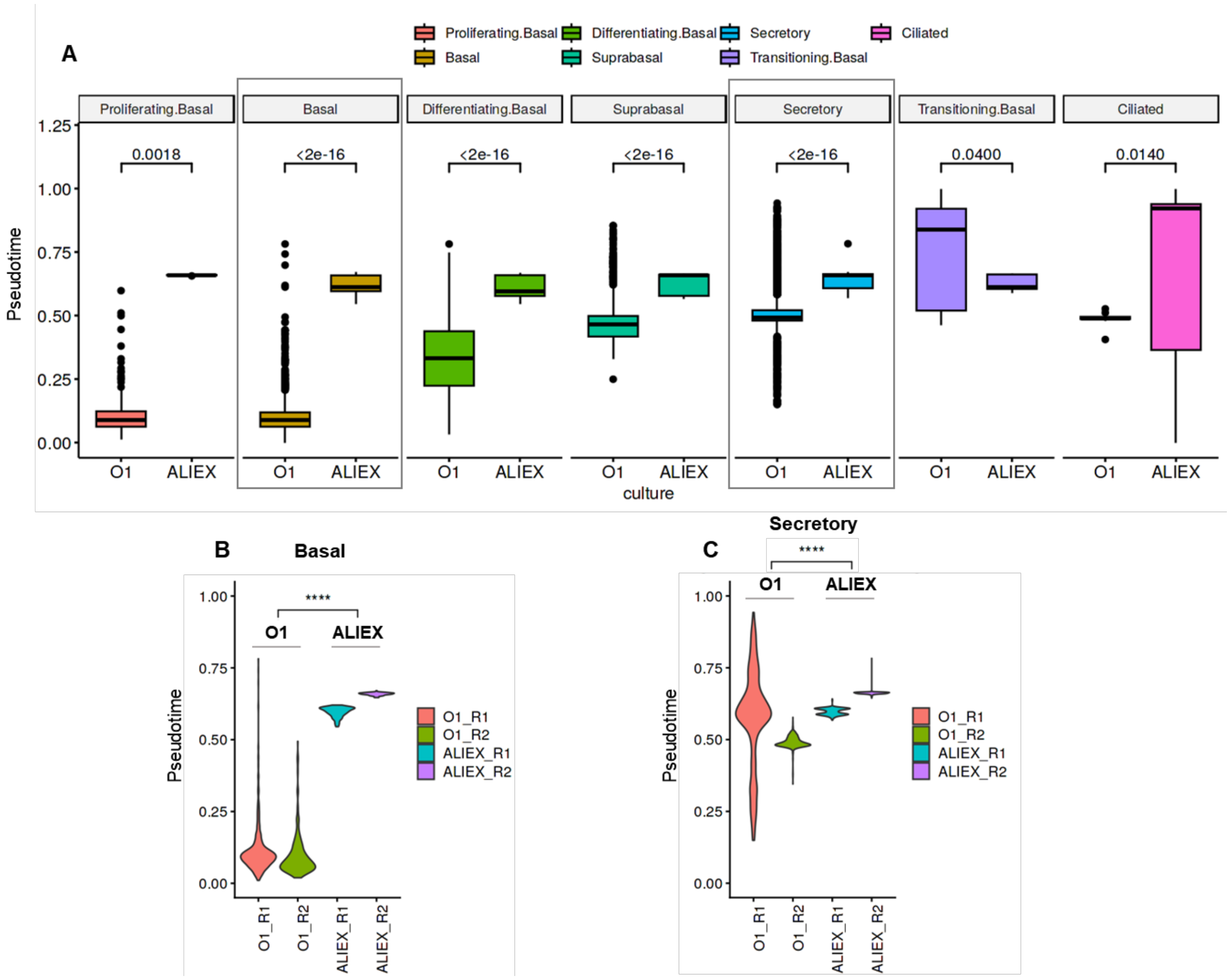

Supplemental Figure S6. Plots of pseudotime values for the ALIEX and O1 models. (**A**) Boxplots of pseudotime for each cell subtype. The *p*-value was adjusted using Holm's method. (**B**) Violin plots of pseudotime in basal cells. (**C**) Violin plots of pseudotime in secretory cells. ****p<0.0001, unpaired t-test.

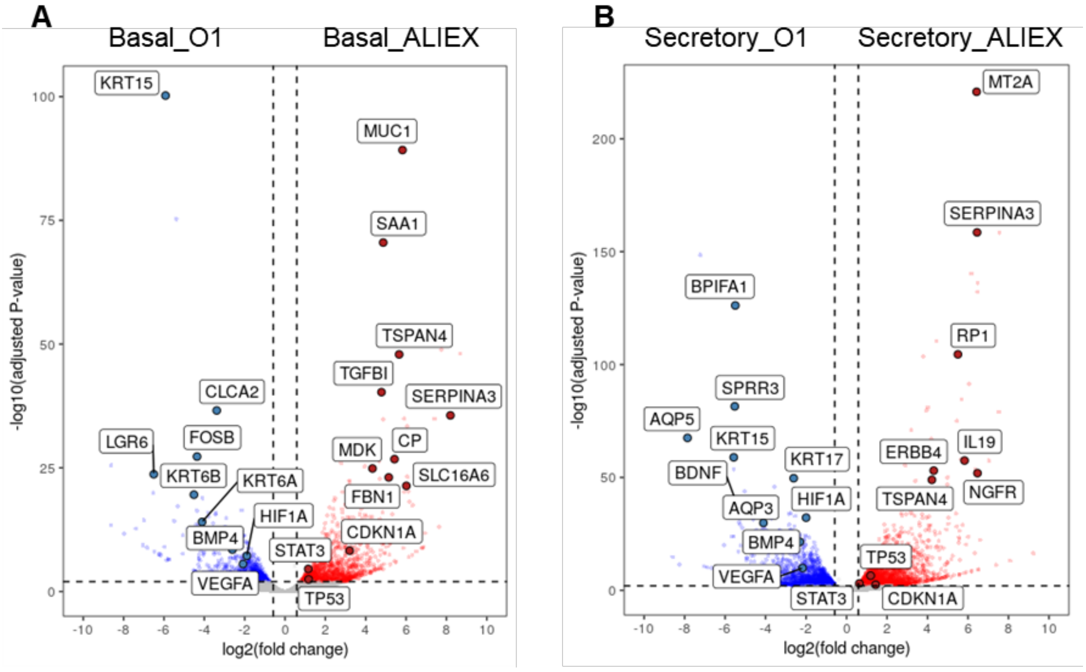

Supplemental Figure S7. Volcano plots of DEGs between the ALIEX and O1 models. The *p*-value was adjusted using the Benjamini–Hochberg method. (**A**) Basal and (**B**) secretory cells.

A

ALIEX vs. O1							
	Basal cells		Secretory cells		Activation state prediction		
Gene	Log2FC	adjp	Log2FC	adjp	Activated p_fdr	Inhibited p_fdr	Predicted as
KDM5B	-0.809	0.008	-0.669	3.E-04	1	0.446	Inhibited
CXCR4	-2.922	0.006	-2.773	0.003	1	0.623	Inhibited
HIF1A	-1.89	1.E-06	-1.999	1.E-06	1	0.636	Inhibited
VEGFA	-2.083	3E-06	-2.173	1.E-06	0.914	0.636	Inhibited
CREB1	-0.872	0.012	-0.686	0.004	0.754	1	Activated
CASP3	-1.301	2.E-04	-1.262	1.E-06	1	1	Unpredicted
BMP4	-2.608	1.E-06	-2.307	1.E-06	1	1	Unpredicted
CDKN1A	3.201	1.E-06	1.435	0.003	1	1	Unpredicted
EPHA2	-1.326	0.009	-2.351	1.E-06	No prediction	No prediction	Unpredicted

B

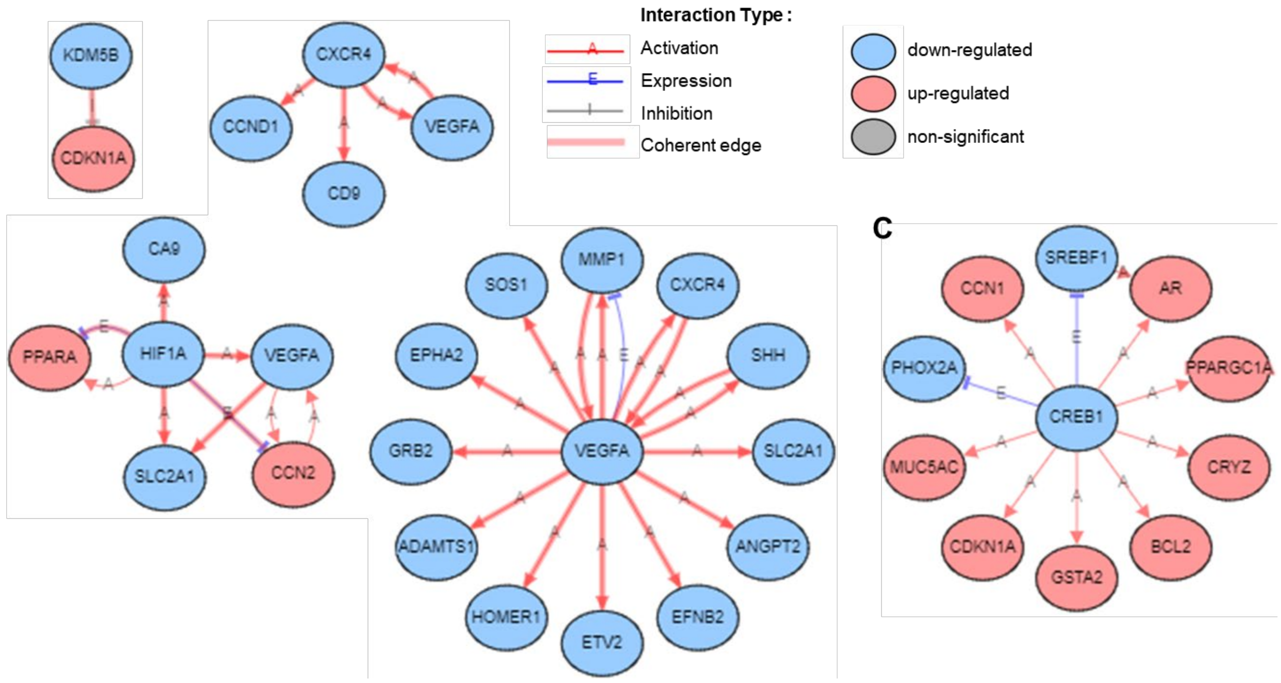

Supplemental Figure S8. Gene activity predictions using the downstream genes. (**A**) Predicted activation states and measured changes in expression of nine genes (*VEGFA, CDKN1A, HIF1A, BMP4, CXCR4, EPHA2, CASP3, KDM5B,* and *CREB1*). (**B**) Coherent expression changes in the downstream genes *KDM5B, CXCR4, HIF1A,* and *VEGFA*. (**C**) Changes in the expression of genes downstream of *CREB1*.

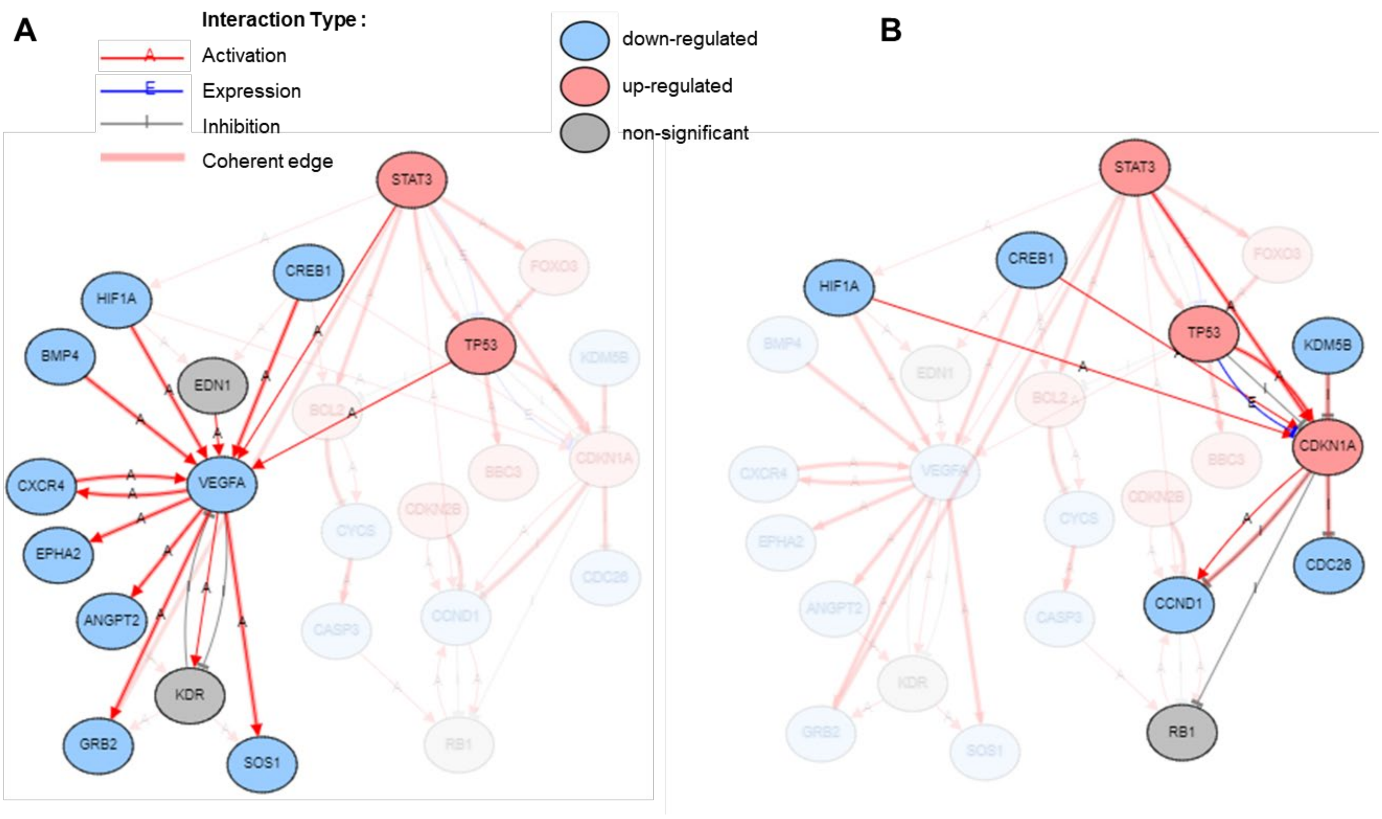

Supplemental Figure S9. Two major streams were identified in the extended gene regulatory network (GRN) (Figure 5A). (**A**) Down-regulated pathway involving *HIF1A*/*BMP4-VEGFA*. (**B**) The upregulated pathway leading to *STAT3-TP53-CDKN1A.*
